# Recovery from stolbur disease in grapevine involves changes in sugar transport and metabolism

**DOI:** 10.3389/fpls.2013.00171

**Published:** 2013-06-04

**Authors:** Simonetta Santi, Federica De Marco, Rachele Polizzotto, Simone Grisan, Rita Musetti

**Affiliations:** Department of Agricultural and Environmental Sciences, University of UdineUdine, Italy

**Keywords:** callose, cell wall invertase, grapevine, recovery, stolbur, sucrose transporters, sucrose synthase

## Abstract

Grapevine can be severely affected by phytoplasmas, which are phytopathogenic *Mollicutes* invading the sieve elements of the host plant. The biochemical and molecular relationships between phytoplasmas and their hosts remain largely unexplored. Equally unknown is an interesting aspect of the pathogen–plant interaction called “recovery,” which is a spontaneous remission of symptoms in previously symptomatic plants. Recovered plants develop resistance mechanisms correlated with ultrastructural and biochemical changes in the sieve elements. Callose as well as sugars are involved in several plant defense processes and signaling. In the present work we have examined the possible involvement of callose, as well as callose synthase, sugar transporter, and cell wall invertase genes, during the infection and after “recovery” of grapevine from bois noir (BN). Ultrastructural investigation of leaf tissue showed that callose accumulated in the sieve elements of diseased grapevine; moreover, two genes encoding for callose synthase were up-regulated in the infected leaves. Regarding sucrose, expression analysis showed that sucrose transport and cleavage were severely affected by BN phytoplasma, which induced the establishment of a carbohydrate sink in the source leaf, and was analogous to other obligate biotrophs that acquire most of their nutrients from the host plant. Interestingly, whereas in recovered plants the transcript level of sucrose synthase was similar to healthy plants, sucrose transporters as well as cell wall invertase were expressed to a greater degree in recovered leaves than in healthy ones. Recovered plants seem to acquire structural and molecular changes leading to increases in sucrose transport ability and defense signaling.

## INTRODUCTION

Phytoplasmas have been associated with several hundred diseases affecting economically important crops, such as ornamentals, vegetables, fruit trees, and grapevines (Lee et al., 2000). Phytoplasmas are plant-pathogenic prokaryotes belonging to the class *Mollicutes*, a group of wall-less microorganisms phylogenetically related to low G+C, Gram-positive bacteria (Weisburg et al., 1989). In host plants, they are restricted to the sieve elements of phloem and are transmitted among plants by phloem sap feeding leafhoppers or psyllids in a persistent manner. Phytoplasmas remain as the most poorly characterized plant pathogens, primarily because efforts at *in vitro* culture, gene delivery and mutagenesis have been unsuccessful.

Bois noir (BN) is a grapevine disease associated with the presence of phytoplasmas of the stolbur group, 16SrXII, known as “*Candidatus* Phytoplasma solani” (“*Ca*. P. solani”), but still not described (Firrao et al., 2005). BN is often endemic, but, in some cases, can be associated with severe epidemics, as reported in several Italian regions in the past 15 years (Belli et al., 2010). BN causes symptoms such as abnormal lignification of canes, short internodes, flower abortion, and curling and discoloration of leaves with intervein yellowing or reddening, and these are accompanied by a dramatic reduction in yield (Osler et al., 1993). These symptoms have been related to stoma closure, reduced photosynthesis rate, and anomalous accumulation of carbohydrates in leaves (Bertamini et al., 2002; Musetti et al., 2007; Endeshaw et al., 2012).

Phytoplasmas are restricted mainly to sieve elements, where they move through the pores of sieve plates and accumulate especially in source leaves and to a lesser extent in petioles and stems (Christensen et al., 2004). Phytoplasmas induce characteristic symptoms in host plants, many of which point to impairment of sieve-tube function (such as low productivity, stunting, general decline, reduced vigor; Kartte and Seemüller, 1991). Cytological modifications such as sieve-element necrosis, abnormal callose deposition at the sieve plates, sieve-element wall thickening, and starch accumulation in the shoots of susceptible plants have been documented by electron microscopic observations (Braun and Sinclair, 1978; Kartte and Seemüller, 1991; Musetti et al., 1994; Musetti and Favali, 1999). During phytoplasma infection, assimilate translocation in the host plant is severely impaired, being responsible for massive changes in phloem physiology (Musetti et al., 2013). It was postulated that phytoplasmas secrete a variety of effector proteins that interfere with plant processes, leading to changes in plant development and physiology, but to date the biochemical and molecular relationships between phytoplasmas and their hosts remain largely unexplored. Global transcription profiles have been obtained from phytoplasma-infected grapevine leaves by hybridization of microarrays (Albertazzi et al., 2009; Hren et al., 2009a) or protein profiling (Margaria and Palmano, 2011). These studies revealed that phytoplasma infection in grapevine altered the expression of more than one hundred genes and thirty proteins belonging to both primary and secondary metabolism. Inhibition of several Calvin-cycle enzyme genes was shown and explained by the accumulation of soluble carbohydrates and starch that had been observed in source leaves of plants infected by phytoplasmas (Lepka et al., 1999; Maust et al., 2003; André et al., 2005). On the other hand, genes encoding enzymes involved in hexose production from sucrose and starch, like vacuolar invertase, sucrose synthase (SUS), and alpha amylase, were shown to be up-regulated (Albertazzi et al., 2009; Hren et al., 2009a,b). A major effect on sucrose transport and metabolism has been confirmed thanks to gene expression analysis focused at the leaf phloem of stolbur-infected grapevine. Laser microdissection-assisted isolation of phloem transcripts and gene expression analysis showed inhibited sucrose loading and increased sucrose cleavage, suggesting the establishment of a phytoplasma-induced switch from a carbohydrate source to sink (Santi et al., 2013).

An interesting but still unclear aspect of the phytoplasma–plant interaction is “recovery,” which is a spontaneous remission of symptoms in previously symptomatic plants, occurring also in BN-infected grapevines (Osler et al., 1993), where the remission of symptoms is associated with the disappearance of the phytoplasmas from the crown as also observed in apple trees recovered from Apple Proliferation disease (Osler et al., 2000; Carraro et al., 2004). Cytochemical analyses have revealed that recovery is accompanied by biochemical changes in the phloem, related to variation of the sieve-element oxidative status, leading to modifications in phloem protein (P-protein) conformation and in phloem occlusion expression patterns. In particular, in grapevines as well as in apple and apricot trees it has been demonstrated that recovery coincided with the accumulation of hydrogen peroxide in sieve elements (Musetti et al., 2004, 2005, 2007), which often signals increased resistance. Moreover, an anomalous accumulation of callose and protein associated with the up-regulation of callose synthase- and P-protein-coding genes has been observed in recovered apple trees (Musetti et al., 2010), supporting the hypothesis that recovered plants were able to develop resistance mechanisms dependent on Ca^2^^+^ signal activity (Musetti et al., 2010, 2013).

Callose is a structural component of the sieve elements (Ehlers et al., 2000; van Bel et al., 2002). Its activity in sieve-pore occlusion in the case of injuries (wounding, pathogen challenge, attack by phloem sap-sucking insects), is extensively reported (Knoblauch and van Bel, 1998; Furch et al., 2007) but roles in sieve element physiology are also recognized (Barratt et al., 2011; Xie et al., 2011). In intact phloem tissue, callose is involved in the correct functioning and development of the sieve elements and in flow regulation through the sieve pores (Barratt et al., 2011; Xie et al., 2011).

In this work we studied the responses (in term of morphological conformation and gene expression analyses) of callose during phytoplasma infection in grapevines affected by BN and their possible role in the establishment of “recovery.” Moreover, the expression of sucrose metabolism-related genes such as sucrose transporters, SUS, and invertase genes were also investigated in leaves of recovered plants compared to infected and healthy plants.

## MATERIALS AND METHODS

### PLANT MATERIAL AND PHYTOPLASMA DETECTION

Grapevines (*Vitis vinifera* L. cv. Chardonnay) were grown in an experimental field located in Friuli Venezia Giulia (North-Eastern Italy). Plants were regularly treated with fungicides. Fully expanded, intact leaves were collected from five healthy (H), five symptomatic (D), and five recovered (R) plants in late summer (five leaves for each plant), when typical BN symptoms were evident. Leaves were collected from symptomatic canes of infected plants, and in similarly positioned canes of nearby recovered and healthy plants. For stolbur phytoplasma detection, RNA was extracted from frozen H, D, and R leaves enriched in midribs using RNeasy Plant Mini Kit (Qiagen GmbH, Hilden, Germany) with minor modifications (Santi et al., 2013). Total RNA quantity and purity were evaluated using a NanoDrop ND-1000 UV–Vis Spectrophotometer (Thermo Fisher Scientific, Inc., MA, USA).

RNAs were reverse-transcribed using a QuantiTect Reverse Transcription Kit (Qiagen GmbH, Hilden, Germany) with random hexamers following the manufacturer’s instructions. Real-time PCR reactions were set up with SsoFast EvaGreen Supermix (Bio-Rad Laboratories Co., Hercules, CA, USA) using specific primers designed on the *16SrRNA* gene of “*Ca*. P. solani” (accession no. AF248959) 16Sstol(RT)F2 and 16Sstol(RT)R3 (Martini et al., unpublished results; Santi et al., 2013). Real-time PCR analyses were performed in a CFX96 Real Time PCR Detection System (Bio-Rad Laboratories Co., Hercules, CA, USA), imposing the following standard thermal profile: 98°C for 2 min, followed by 45 cycles for 5 s at 98°C and 5 s at 60°C. A melting curve analysis of the products was performed from 65 to 95°C to check primer specificity.

### TRANSMISSION ELECTRON MICROSCOPY

To minimize the damage to sieve elements due to the electron microscopy preparation procedure, a gentle preparation of the samples has been performed according the method described by Ehlers et al. (2000). Segments (6–7 mm in length) of grapevine leaf tissues including the vein and 1–2 mm of blade on each side were excised, immersed in a buffered medium containing 10 mM NaOH-2-(*N*-morpholino) ethanesulfonic acid, 2 mM CaC1_2_, 1 mM MgC1_2_, 0.5 mM KC1, and 200 mM mannitol, pH 5.7 (van der Schoot and van Bel, 1989), and allowed to recover for 2 h at room temperature. Then, the buffer was replaced by a fixation solution of 3% paraformaldehyde and 4% glutaraldehyde in 50 mM sodium cacodylate buffer plus 2 mM CaCI_2_, pH 7.2.

Samples were fixed for 6 h at room temperature, replacing the fixative every 30 min. Then they were washed for 1 h at 4°C in 50 mM sodium cacodylate buffer containing 2 mM CaCl_2_ (pH 7.2) and postfixed overnight with 2% (w/v) OsO_4_ in the above buffer at 4°C.

Dehydration was performed in a graded ethanol series and propylene oxide, and the samples were embedded in Epon/Araldite epoxy resin (Electron Microscopy Sciences, Fort Washington, PA, USA).

Several serial ultrathin sections of at least 100 samples from each of the three plant groups were collected on copper grids, stained in uranyl acetate and lead citrate and observed under a PHILIPS CM 10 (FEI, Eindhoven, The Netherlands) transmission electron microscope (TEM) operating at 80 kV.

### PLANT GENE EXPRESSION ANALYSES

For plant gene expression analyses, RNAs were extracted from frozen leaf midribs as described above. Nucleic acids were treated with Turbo DNase (Ambion, Life technologies Co., Carlsbad, CA, USA) and reverse-transcribed using a SuperScript III Platinum Two-Step qRT-PCR Kit (Invitrogen Life Technologies, Paisley, UK) in a total volume of 20 μL. Real-time RT-PCR analyses were performed on a CFX 96 instrument (Bio-Rad Laboratories Co., Hercules, CA, USA). SsoFast EvaGreen Supermix (Bio-Rad Laboratories Co., Hercules, CA, USA) was used for the analysis of callose synthases, while RealMasterMix SYBR ROX (5Prime Eppendorf, Hamburg, Germany) for all other genes. In both cases a melting curve analysis was performed from 65 to 95°C to check primer specificity. Primers were designed at 60°C Tm using Primer3 software^1^ , and then evaluated using the BLASTN (nucleotide basic local alignment search tool) algorithm (Altschul et al., 1997). Standard curves of different dilutions of pooled complementary DNA (cDNA) were used to calculate the PCR efficiency value (E) for each primer pair as described by Pfaffl (2001). Primers and E are indicated in **Table 1**.

**Table 1:** Accession number of sequences and primers used for real-time RT-PCR.

Gene name	NCBI accession No.	Primer sequence (5′–3′)	nM E	Grape Genome 12X Locus tag
***VvUBQ-L40***	XM_002273532.1	For: CCAAGATCCAGGACAAGGAA Rev: GAAGCCTCAGAACCAGATGC	300 2.05	GSVIVT01038617001
***VvSUC11***	AF021808.1	For: ATGGAGAAGCTCTGCAGGAA Rev: TCAGTGCAGCAATCACAACA	300 1.93	GSVIVT01009254001
***VvSUC12***	AF021809.1	For: CGGATTGGATGGGTAGAGAA Rev: AGCAAACCAAATGCACCTTC	500 1.91	GSVIVT01020031001
***VvSUC27***	AF021810.1	For: CTCTTCGACACCGATTGGAT Rev: CAACCCCAGAACCACAGAGT	300 1.97	GSVIVT01034886001
***VvSUS4***	XM_002275119.1	For: GCTGGCTCAATCAGTTCCTC Rev: CCAAGCCTCAAACAATGACA	500 2.01	GSVIVT01015018001
***VvSUS2 ***	XM_002271860.1	For: GGCTGGGGTTTATGGTTTCT Rev: ATTTTGCCAGATCACGGAAC	300 2.06	GSVIVT01028043001
***VvSUS6***	XM_002270825.1	For: TATGGCTTCTGGAGGCAGTT Rev: CCTTCGCCAATTTTCTGAAT	500 2.02	GSVIVT01029388001
***VvSUS5***	XM_002266984.2	For: GCAGGGATGATTCAGACCAT Rev: CTTGCTTGTGTTCCGTGTTC	300 2.07	GSVIVT01035210001
***VvGIN2***	XM_002272733.1	For: ACGAATTTTTGGGAGCACAG Rev: GATGCATGTCCTTCCACCTT	500 2.05	GSVIVT01001272001
***CWINV***	AY538262.1	For: TATTGACGGTGAAGCCCACT Rev: AAAGCCTGGCTCTTCACTCA	300 1.99	GSVIVT01016869001
***VvGIN1***	XM_002265498	For: CAATGCCACTGGAGTGAATG Rev: GGGATTTCTCAGCAACCAAA	500 1.94	GSVIVT01018625001
***VvCAS2***	XM_002283262.2	For: TTCACCCCAGTTGCATTTCT Rev: CCGATCCTTCCTATGACCAC	300 2.05	GSVIVT01025362001
***VvCAS1***	XM_002271612.2	For: GCCTTGCGCTTTTTCATCTA Rev: CTTCGCCTTCCAACAGAGAG	300 2.01	GSVIVT01001361001
***VvCAS7***	XM_002279310.2	For: GCTGGGAAGGGCTTATGAGT Rev: GGCCTCTACTGAATGCCTGA	300 2.01	GSVIVT01020854001
***VvCAS11***	XM_002263721.2	For: GCTGAACAGAGCTGGGAAAC Rev: CGCCGTACTGGAAAAAGAAG	300 1.97	GSVIVT01005204001
***VvCAS5***	XM_002274301.1	For: GCATGGTTCCCATTTGTCTC Rev: TCATCACAGCCTCACTCTGC	300 1.96	GSVIVT01026489001
***VvCAS10***	XM_002275082.2	For: GATGCTGGGATGGGTATGAT Rev: CCTGCAAGGATGATGGAGAT	300 2.03	GSVIVT01007560001

Ubiquitin-60S ribosomal protein L40-like (*UBQ-L40*; accession no. XM_002273532.1) was used as a reference gene, as it was found to be stably expressed when compared with actin, *UBQCF* (Ubiquitin conjugating factor) or *60SRP* (60S ribosomal protein L18). The gene-stability measure (M) was calculated by the geNorm program (Vandesompele et al., 2002) using RNAs that were purified from midrib-enriched-samples of several D, R, and H plants. The M value for *UBQ-L40* was 0.45, thus confirming the validity of this gene as a reference. A mean normalized expression (MNE) for each target gene (Muller et al., 2002) was calculated by normalizing its mean expression level to the level of ubiquitin, with the transcript abundance of ubiquitin defined as 100 arbitrary units. Mean normalized gene expression values were graphed by assigning a value of zero to no expression. Each data point represents the mean of at least three biological replicates and three technical replicates. The sequences of the examined *V. vinifera* genes were identified *in silico* by Grape Genome browser^2^ or retrieved from the National Center for Biotechnology Information (NCBI) database.

Statistical analyses of gene expression levels were performed with the InStat GraphPad software package (La Jolla, CA, USA) using an ANOVA Tukey–Kramer Multiple Comparisons Test.

## RESULTS

### PHYTOPLASMA DETECTION

Leaf samples were analyzed for the presence of BN phytoplasma by real-time RT-PCR. Starting from 10 ng of total RNA, stolbur *16SrRNA* transcripts were detected in symptomatic samples (D) at an average quantification cycle (Cq) value (±SE) of 24.0 ± 0.5, while no amplicons of the *16SrRNA* gene were obtained in healthy (H) and recovered (R) samples. Molecular diagnosis confirmed the presence of stolbur in 98% of leaf samples collected from plants classified as symptomatic in the field.

### TRANSMISSION ELECTRON MICROSCOPY

**FIGURE 1:**
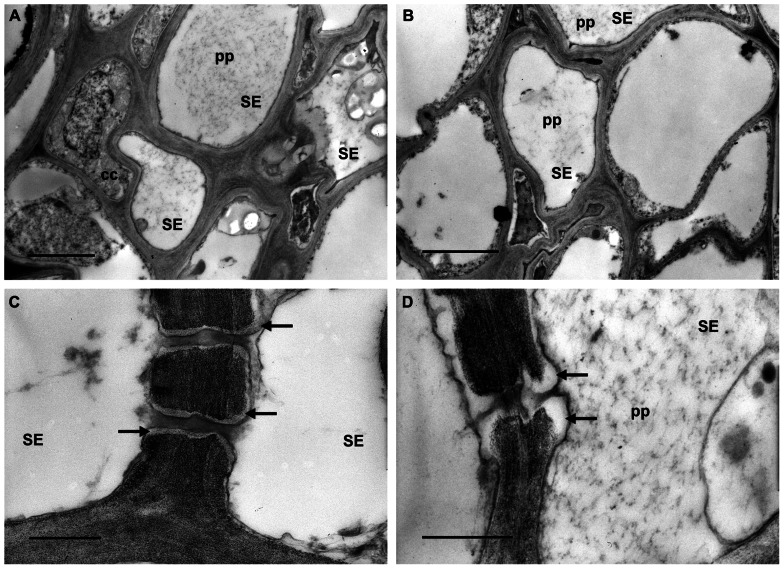
**Transmission electron micrographs of leaf tissues from healthy grapevines**. **(A,B)** P-protein (pp) is uniformly dispersed in the lumen of most sieve elements (SE). **(C,D)** Sieve pores are surrounded by a thin callose layer (**C**, arrows) or show callose collars that do not occlude their lumen (**D**, arrows). In **(A)** and **(B)** bars correspond to 2 μm; in **(C)** and **(D)** bars correspond to 0.5 μm.

Transmission electron microscopy investigations showed different ultrastructural traits in the phloem of the grapevines, depending on their status (H, D, or R). The leaf tissues from H plants were well preserved. As expected, in the sieve elements of these plants phytoplasmas were not detected. P-protein was uniformly dispersed in the lumen of most sieve elements (**Figures 1A,B**, pp) and sieve pores were surrounded by a very thin stratum of callose (**Figure 1C**, arrows) or had callose collars that did not occlude their lumen (**Figure 1D**, arrows).

**FIGURE 2:**
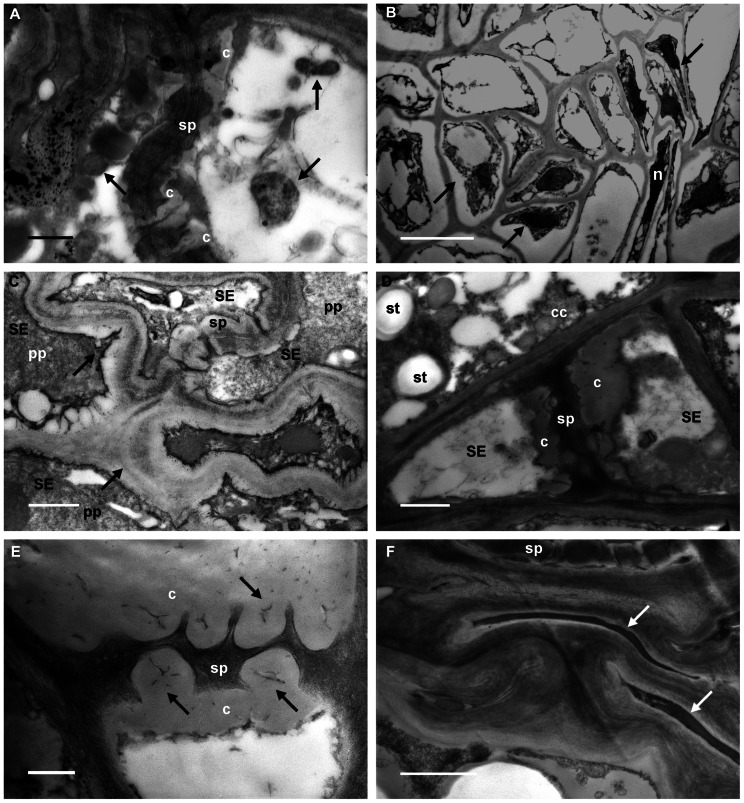
**Transmission electron micrographs of leaf tissues from diseased grapevines**. **(A) **Phytoplasmas are visible in the lumen of sieve elements (arrows). Callose (c) is accumulated at the sieve plate (sp). **(B)** Phloem parenchyma and companion cells show plasmalemma detachment from the cell wall and condensed cytoplasm (arrows). Some cells are necrotized (n). **(C) **Sieve-element (SE) walls appear increased in thickness (arrows) and P-protein filaments (pp) are organized in dense plugs (sp: sieve plate). **(D,E)** A big accumulation of callose (c) at the sieve plates (sp), and occluding the sieve pores (**E**, arrows), is visible in infected samples. Starch granules (**D**, st) are detectable in the companion cell (cc; SE, sieve elements). **(F)** Groups of collapsed cells are present in the phloem of infected grapevine leaves (arrows); at the top, a sieve plate (sp) is still recognizable. In **(A)** and **(E)** bars correspond to 0.5 μm; in **(B)** bar corresponds to 5 μm. In **(C)**, **(D)**, and **(F)** bars correspond to 1 μm.

In D leaf tissues phytoplasmas were detected in the lumen of sieve tubes (**Figure 2A**, arrows). Their presence is associated with severe ultrastructural modifications of the phloem (**Figures 2B** through F). Many companion and phloem parenchyma cells showed plasmolysis and consequent cytoplasm condensation (**Figure 2B**, arrows), others were necrotized (**Figure 2B**, n). Increased thickness of the sieve-element walls (**Figure 2C**, arrows) and a large accumulation of callose at the sieve plates, plugging the sieve pores (**Figures 2D,E**, c), were also visible. Sieve elements were often collapsed (**Figure 2F**, arrows). Given the serious ultrastructural disorganization of D grapevine leaf tissues, in many cases it was not possible to discern P-proteins in the sieve-element lumen. When detectable, P-protein filaments were organized in electron-dense clumps (**Figure 2C**, pp).

**FIGURE 3:**
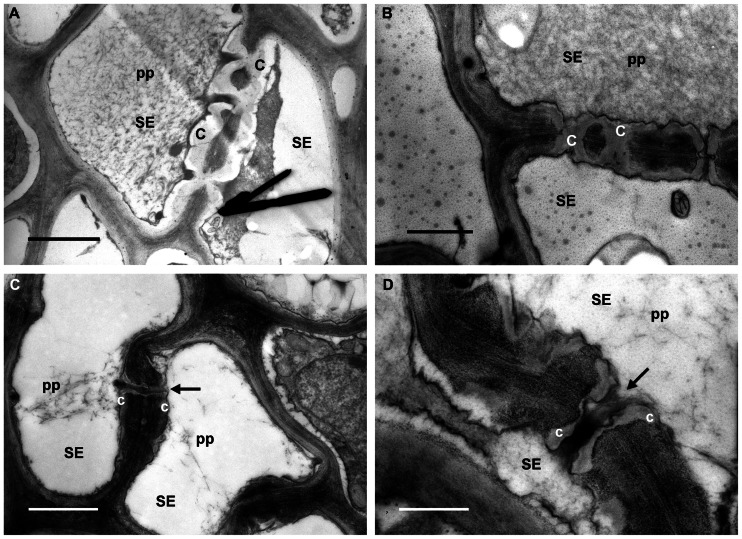
**Micrographs of leaf tissue from recovered grapevines**. **(A,B)** P-protein (pp) in condensed form is present in the sieve-element (SE) lumen and also in association with callose collars around the sieve pores (c). **(C,D)** P-protein filaments (pp) are localized in the proximity of the sieve plate, and were likely passing through the sieve pores (arrows). Note the thin callose layers (c) surrounding the sieve pores (arrows). In **(A)**, **(B)**, and **(C)** bars correspond to 1 μm; in **(D)** bar corresponds to 0.5 μm.

In R plants the leaf tissue was, in general, well preserved and phytoplasmas were not observed in the sieve elements (**Figure 3**). P-protein was observed in the sieve-element lumen as condensed plugs (**Figures 3A,B**, pp) or as filaments (**Figures 3C,D**, pp) likely passing through the sieve pores (**Figures 3C,D**, arrows). Callose was also found in sieve tubes of R plants, forming collars around the sieve pores (**Figures 3C,D**, c ), and in some cases occluding them (**Figures 3A,B**, c ).

### CALLOSE SYNTHASE GENE EXPRESSION ANALYSIS

**FIGURE 4:**
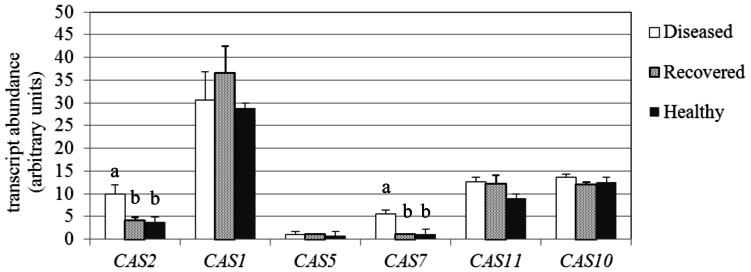
**Gene expression analysis of*Vitis vinifera* callose synthase (CAS) genes.** Mean expression (MNE) values from at least three individuals for each plant group (H, D, R) plus standard errors are shown. Expression levels of the genes of interest are normalized to ubiquitin (*UBQ-L40* expression level = 100). Statistical comparisons were made using an ANOVA Tukey–Kramer Multiple Comparisons Test to evaluate significant differences. When indicated, different letters denote significant differences (*P* < 0.05).

Callose synthases synthesize the β-1,3-glucan (callose) that accumulates at the sieve plates. Callose is usually deposited at plasmodesmata and at sieve plates as a response to developmental cues and pathogen attack, with the aim of limiting spread of the infection or reinforcing the cell wall (Nakashima et al., 2003). The *Arabidopsis* genome contains 12 callose synthases (CalS), also known as glucan synthase-like genes (GLS), which are each expressed in a tissue- and developmental stage-specific manner, as well as in response to different physiological and environmental inducers (Verma and Hong, 2001). Among seven genes encoding for callose synthases (called *CAS* in this work) that were identified from the Grape Genome browser^3^ in the Grape 12X genome Database, six were expressed in leaves of D, R, and H plants, and only two were up-regulated in D leaves; *VvCAS2* (accession no. XM_002283262.2) and *VvCAS7* (accession no. XM_002279310.2; **Figure 4**). The expression levels of *VvCAS2* and *VvCAS7* were 9.9 and 5.5 MNE units, respectively, in D leaves, whereas the levels were 3.8 and 1.2, respectively, in H leaves. H and R samples did not show significant differences in *VvCAS2* and *VvCAS7* transcript levels (**Figure 4**). *VvCAS1* (accession no. XM_002271612.2) was the most highly expressed in leaves, and was not even differentially modulated in response to infection or after recovery, similar to the other expressed CAS isogenes (accession numbers and primers are reported in **Table 1**).

The CAS member of the family called *CAS8-like* (accession no. XM_002267920.2) showed a transcript level lower than 0.5% compared to the reference gene in all samples and is not shown.

### EXPRESSION ANALYSIS OF GENES FOR SUCROSE TRANSPORT AND METABOLISM

**FIGURE 5:**
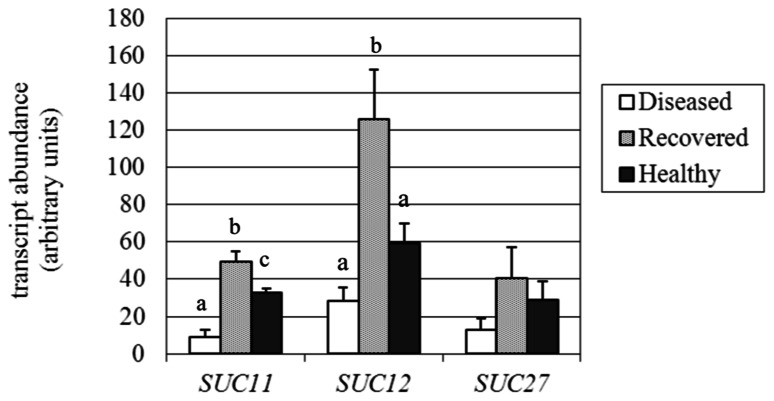
**Gene expression analysis of*Vitis vinifera* sucrose transporter (SUC) genes.** Mean expression (MNE) values from at least three individuals for each plant group (H, D, R) plus standard errors are shown. Expression levels of the genes of interest are normalized to ubiquitin (*UBQ-L40* expression level = 100). When indicated, different letters denote significant differences (*P* < 0.05).

**FIGURE 6:**
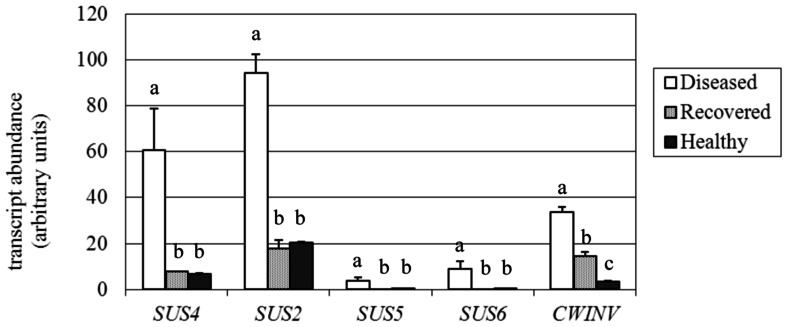
**Gene expression analysis of*Vitis vinifera* sucrose synthase (SUS) and cell wall invertase (cwINV) genes**. Mean expression (MNE) values from at least three individuals for each plant group (H, D, R) plus standard errors are shown. Expression levels of the genes of interest are normalized to ubiquitin (*UBQ-L40* expression level = 100). When indicated, different letters denote significant differences (*P* < 0.05).

Expression analysis of genes related to sucrose transport and metabolism was performed in midrib-enriched, fully expanded intact leaves of infected, recovered and healthy grapevines by real-time RT-PCR (**Figures 5** and **6**). In grapevines, as in most plants, sucrose is the carbohydrate for long distance transport, and glucose and fructose are the major soluble sugars that accumulate in sink organs like berries (Coombe, 1992). Accumulation of glucose and fructose is mainly attributed to three families of proteins: the sucrose transporters (Sauer, 2007), the acid (vacuolar or cell wall-associated) and neutral (cytosolic) invertases, and the SUSs (Koch, 2004).

The expression of the three sucrose transporter genes (*SUC27, SUC11*, and* SUC12*; Davies et al., 1999) investigated in the present work (accession numbers and primers are reported in **Table 1**) was confirmed as being decreased in D leaves, as already observed by Santi et al. (2013). *SUC12* was the most highly expressed transporter in the H leaves, but was down-regulated more than twofold in D samples, although the high variability among individuals negatively affected the significance of the difference (**Figure 5**). Our analysis was extended to the recovered plant leaves, where *SUC12* was more than fourfold (125.9 vs 28.4 MNE units) and twofold (125.9 vs 59.2 MNE units) higher than in D and H samples, respectively, due to it being highly affected during recovery from the pathogen. The *SUC12* gene belongs to the type IIA cluster of sucrose transporters (Reinders et al., 2012), which include the *Arabidopsis* *SUT2/SUC3* gene. A role for *SUC12* in sucrose unloading into grapevine berry tissues was proposed by Afoufa-Bastien et al. (2010). In accordance with this role, *SUC12* was almost undetectable in phloem cells (including sieve elements, companion cells, and surrounding parenchyma cells) that had been captured by laser microdissection from source leaves of grapevine (Santi et al., 2013). Similarly, *SUC11* expression was shown to be 3.5-fold inhibited in D leaves (8.9 vs 32.9 MNE units) compared to H leaves, while it was up-regulated in R leaves, where the transcript level (49.3 MNE units) was more than fivefold higher than in D leaves (**Figure 5**). The grapevine* SUC11* belongs to the type III group of low affinity sucrose transporters and its *Arabidopsis* ortholog is *ATSUC4* (Reinders et al., 2012). *ATSUC4* is localized at the tonoplast of *Arabidopsis* mesophyll cells (Endler et al., 2006). *SUC11* transcripts were found to be expressed at a very low level in the phloem tissue of source leaves (Santi et al., 2013). When analyzed in R leaves, the *SUC27* gene also seemed to be more induced (threefold) than in D leaves, although the difference between R leaves, and D and H leaves was not significant (**Figure 5**). The *SUC27* gene belongs to the type I cluster of sucrose transporters, including transporters necessary for loading sucrose into the phloem, among which the *Arabidopsis ATSUC2* is also grouped (Reinders et al., 2012). *SUC27* is the only sucrose transporter that is expressed in the phloem tissue of source leaves of grapevine and was seen to be dramatically down-regulated in the presence of stolbur (Santi et al., 2013). The phloem specificity of *SUC27* gene expression and thus the dilution of its transcripts by functionally different tissues could explain why the differences observed in the leaf samples were not significant. In conclusion, the examined *SUC* genes confirmed an overall expression decrease in stolbur-infected leaves, even if to a different extent, while they were up-regulated during the recovery from BN compared to H leaves.

Sucrose synthase has a dual role in directing carbon to polysaccharide biosynthesis and in conserving energy throughout the production of adenylated-glucose (Winter and Huber, 2000; Koch, 2004). Its cleavage activity produces fructose and both uridine diphoshate-glucose (UDPG) and adenosine diphosphate-glucose (ADPG); ADPG is necessary for starch biosynthesis, while UDPG is necessary for cell wall and glycoprotein biosynthesis (Baroja-Fernández et al., 2012). UDPG is also used by callose synthase (CAS) as a glucose donor to the growing polymer chain (Amor et al., 1995). Among *SUS* isoforms (at least five in grapevine), four were found to be expressed in leaves and thus were investigated by real-time RT-PCR (accession numbers and primers are reported in **Table 1**). Transcripts of a fifth *SUS* (accession no. XM_002271494) were almost undetectable in leaves and are thus not shown. Although expressed at different levels in H leaves (**Figure 6**), all the examined SUS genes were significantly up-regulated in the presence of the pathogen, as previously observed just for the *VvSUS4* gene (named* VvSUS2-like* in Santi et al., 2013). The analysis of leaves from plants recovered from infection showed the expression of all the investigated *SUS* genes at the level of healthy leaves. The most expressed SUS in H and R leaves was *VvSUS2* (about 19 MNE units), which was 4.7-fold induced in D leaves. This gene of the family (annotated as *SUS2* with the accession no. XM_002271860.1 in the NCBI database) shares 82 and 79% identity (at the amino acid level) with *ATSUS3* (AT4G02280.1) and *ATSUS2* (AT5G49190.1), respectively. In addition, *VvSUS4* was investigated by real-time RT-PCR in leaves where it had increased approximately ninefold in response to stolbur infection (60.8 vs 6.6 and 7.9 MNE units in H and R leaves). This gene shares 82 and 81% identity at the level of amino acidic sequence with *Arabidopsis* *SUS4* (AT3G43190.1) and *SUS3*, respectively (Bieniawska et al., 2007), and was found to be highly affected by stolbur alongside the *SUC27* transporter in phloem cells (Santi et al., 2013). Two other *SUS* genes were examined, *VvSUS5* and *VvSUS6*; although barely expressed in H and R leaves, they were both significantly up-regulated in D leaves like the other SUS genes.

Accumulation of glucose and fructose in grapevine is mainly attributed to the cleavage activity of invertases (Davies and Robinson, 1996). The acid soluble vacuolar invertases *GIN1* and *GIN2* (Davies and Robinson, 1996) were investigated. In our experiment *GIN1* and *GIN2* were expressed at a very low level (on average around 0.3 MNE units) both in H and R leaves, and only for *GIN2* was it possible to detect an up-regulation of the gene in D conditions (data not shown). Low expression of *GIN* genes depends on the fact that their transcripts decline during leaf development (Davies and Robinson, 1996) and in our experiments could reflect the use of mature leaves. Therefore, our attention was focused on the expression of the acid cell wall invertase *VvcwINV* (accession no. AY538262.1). We found that the expression of *VvcwINV* was more than ninefold and fourfold induced in stolbur-infected leaves and recovered leaves, respectively, in comparison with healthy leaves (**Figure 6**).

## DISCUSSION

The phloem is the transmission route for photoassimilates in plants, but it is also a preferred destination for plant pathogens because it is a pathway for their movement and spread inside the host.

Phytoplasmas are phloem-restricted pathogens: knowledge about their ability to interact with sieve elements is essential for understanding the relationships with the whole host plant during both the symptomatic and the following “recovery” phases. Given that “recovery” is a natural, spontaneous event, and not reproducible artificially, an explanation of the phenomenon is not simple. However, this phenomenon is pivotal to counteracting phytoplasmas because there are no effective treatments available. This study confirmed that recovered plants are not colonized by phytoplasmas in the canopy, as already reported in literature (Carraro et al., 2004; Musetti et al., 2007), therefore, regarding the epidemiological and practical aspects, recovered individuals exhibit a performance similar to healthy, never infected plants (Osler et al., 2000).

For the first time we have investigated and reported modifications occurring in the sieve elements of grapevine during BN infection and “recovery.” An integrated approach has been adopted through the combined use of ultrastructural and gene expression analyses of leaf tissues.

Ultrastructural observation of phloem tissues is problematic, because the excision of the samples for electron microscope preparation provokes artifacts due to the immediate wounding response in the sieve elements. To minimize this risk, a gentle method for sample preparation was used, as described by Ehlers et al. (2000). The method allowed us to compare the ultrastructure of the sieve elements and the sieve plates in H, D, and R grapevine leaf tissues and observing differences that are mostly related to callose deposition.

Interestingly, the ultrastructural characteristics found in the sieve elements of R plants were not different from those observed in the H plants, apart from callose.

Callose accumulation is one of the major ultrastructural characteristics of sieve elements of D grapevines. Also, in the case of BN-infected grapevines, phytoplasma-induced callose could be responsible for sieve-tube occlusion and mass flow impairment (linked to the expression of typical BN disease symptoms) as recently demonstrated in a different plant/phytoplasma interaction (Musetti et al., 2013).

Callose depositions in sieve elements of R plants appeared thicker than those observed in H grapevines and, only in some sporadic cases, they occluded the pores of the sieve plates. It seems that these ultrastructural characteristics are compatible with the correct physiology of R plants that are asymptomatic and look identical to H plants. It has been demonstrated that callose deposition in the phloem is not only associated with responses to wounding or to pathogen spread but also that it takes part in the normal processes of phloem maturation in intact plants, influencing the length of the sieve-plate pores and determining the flow characteristics (Barratt et al., 2011; Xie et al., 2011).

Ultrastructural observations about callose deposition in grapevine are in accordance with the results obtained from the gene expression analyses, demonstrating that at least two different isoforms of callose synthase genes are triggered in grapevine during phytoplasma infection and that they return to a lower level (not different from the H plants) during the “recovery” phase.

Most genes encoding CAS, identified in several plant species, are members of multigene families (Verma and Hong, 2001). Multiple *CAS* genes may have evolved in higher plants for callose synthesis at different locations and in response to different physiological and developmental signals (Chen and Kim, 2009). Six *CAS* genes were expressed in the leaves of H, D, and R grapevines. One of these, *VvCAS7*, which is an ortholog of the *Arabidopsis CalS7* that is responsible for callose deposition in the phloem (Xie et al., 2011), was significantly up-regulated in D leaf tissues compared to H and R plants, in accordance with observations by TEM. The second up-regulated gene in D grapevines was *VvCAS2*, which is induced when its expression is examined in the whole leaf (as previously reported by Hren et al., 2009a) but not affected by stolbur in the phloem (Santi et al., 2013).

Our results suggested that at least two CAS genes could be coordinately expressed with SUS genes, which were up-regulated in D leaves but showed a similar expression level in R and H leaves. As the synthesis of callose requires several steps, and hence a single peptide may not be able to perform all the necessary functions, the existence of a callose synthase complex has been suggested (Verma and Hong, 2001). It has been demonstrated that the callose synthase complex has a transmembrane domain and hydrophilic loop interacting with different proteins, related to sucrose synthesis and metabolism, among which SUS is included (Verma and Hong, 2001). The function of some of these proteins may be involved in controlling CAS activity, particularly in response to biotic/abiotic signals (Verma and Hong, 2001). At confirmation of the link between CAS and SUS, Barratt et al. (2009) reported that callose at the sieve plates is reduced significantly in *Arabidopsis* double mutants for the phloem-specific *SUS5* and *SUS6.*

CASs are membrane-associated enzymes (Chen and Kim, 2009). Like several plant membrane-associated polysaccharide synthases (among which cellulose synthase, callose synthase, xyloglucan glucan synthase and multiple related glycan synthases) the CASs have the topological requirements to couple synthesis with the transport of callose into the extracellular matrix and thus are included in the group of dual-function cell wall glycan synthases (Davis, 2012).

The anomalous deposition of callose in the infected phloem tissue and the altered modulation of two callose synthase genes were only a part of the large modifications at the transcriptional level observed for sucrose transport and metabolism of the leaf. Accumulations of soluble carbohydrates and starch in source leaves, complemented by a decrease of sugar levels in sink organs, have been reported for periwinkle, tobacco, and coconut palm infected by phytoplasmas (Lepka et al., 1999; Maust et al., 2003). We previously demonstrated that, analogously to other obligate biotrophs that need to acquire most nutrients from the host plant, the stolbur phytoplasma induces the establishment of a carbohydrate sink in the phloem of the leaf, thus altering the normal pattern of sugar partitioning of a source leaf (Santi et al., 2013). In fact, in laser-microdissected phloem tissue of stolbur-infected leaves, we observed a dramatic decrease of expression of *SUC27*, the grapevine transporter mediating sucrose apoplasmic loading into the phloem, and a huge up-regulation of a SUS gene (*VvSUS4*, Santi et al., 2013). A co-regulation of both sucrose transport and cleavage would be advantageous for the pathogen as both responses are crucial to access hexoses, which could be the only usable carbon source (Christensen et al., 2005). In the present work we analyzed the expression of the *SUC* genes together with four *SUS* genes and the cell wall invertase gene, *cwINV*, in midrib-enriched leaves of D and H plants, extending our investigation to leaves of plants recovered from the disease. Even though they changed to a different extent, all the *SUC* genes were down-regulated in D leaves as expected, confirming that the stolbur-induced establishment of a carbohydrate sink in the phloem alters sucrose partitioning in tissues distal to the infection site. A role for *SUC11* and *SUC12* in sucrose accumulation in the vacuole and in sucrose unloading into sink tissues (berry), respectively, has been suggested by Afoufa-Bastien et al. (2010). In accordance the above finding, all the examined *SUS* genes together with *cwINV* were up-regulated in D leaves.

Sucrose synthase utilizes sucrose to produce fructose and nucleoside diphosphate-glucose (mainly UDPG and ADPG; Winter and Huber, 2000) and thus has a role in producing metabolic substrates, in conserving energy in the form of adenylated-glucose, and in initiating sugar signaling (Koch, 2004). SUS is encoded by a small family of genes that are divergent and differently expressed. The *Arabidopsis* genome contains six SUS genes whose exact function remains unknown because most mutants show few observable effects (Bieniawska et al., 2007). Four grapevine genes were found expressed in leaves, although at a different level, and all were up-regulated in the presence of the phytoplasma confirming the pivotal role of this gene family in both carbohydrate partitioning and plant–pathogen interactions. SUS is also believed to play a major role in both starch and cellulose biosynthesis (Baroja-Fernández et al., 2012). The UDPG produced by a membrane-associated form of SUS is thought to be used directly as a substrate for cellulose synthase in the rosette complex where SUS is an integral component (Amor et al., 1995; Fujii et al., 2010). As discussed above, UDPG is also used as a glucose donor to the growing polymer chain by callose synthases, similar to cellulose synthases.

Cell wall invertase (cwINV), a sucrose-splitting enzyme that produces hexoses, is a sink-specific enzyme, normally found in various kinds of carbohydrate consuming tissues, and its activity is usually low in source leaves. Extracellular invertases are important for apoplastic phloem unloading and are key enzymes in determining sink strength (Roitsch and Gonzàlez, 2004). The inhibition of phloem loading and the induction of SUS genes that were observed in stolbur-infected leaves were expected to be accompanied by elevated cwINV activity, as already observed in *Arabidopsis* (Fotopoulos et al., 2003; Chandran et al., 2010), wheat (Sutton et al., 2007), and grapevine leaves (Hayes et al., 2010) when infected by biotrophic fungi. In the case of grapevine, *VvcwINV* was highly induced in coordination with the hexose transporter, *VvHT5*, by powdery and downy mildew infection. Interestingly, this response, which was also observed with abiotic stress (such as wounding), mirrored the response observed when leaves were treated with ABA (abscisic acid), suggesting the concept that the transition from source to sink following the induction of *cwINV* and *HT* genes is part of a general ABA-mediated stress response (Hayes et al., 2010). It is well established that cwINVs are transcriptionally regulated by hexoses (Roitsch and Gonzàlez, 2004), but interestingly, both ABA-responsive (ABRE) and hexose-responsive (SURE) elements were identified in the promoter of *VvcwINV* (Hayes et al., 2010). It is likely that a coordinated interaction of sugar and hormonal pathways in plants leads to effective immune responses (Bolouri-Moghaddam and Van den Ende, 2012).

It is known that plant cell wall invertases have a pivotal role in plant defense (Roitsch and Gonzàlez, 2004; Berger et al., 2007). Genes encoding cwINV are induced by elicitors in different plant species (Roitsch et al., 2003). Several lines of evidence suggest that plants establish high hexose levels in response to invading pathogens, which in turn support defense responses of the host. Indeed, RNA interference knockdown of cwINV in tobacco leaves inhibited defense responses such as callose deposition, induction of pathogenesis-related proteins, and hydrogen peroxide-mediated cell death against the biotrophic oomycete *Phytophthora nicotianae* (Essmann et al., 2008). Within sugar pools, the cellular sucrose:hexose ratio is emerging as an important parameter determining cellular responses (Bolouri-Moghaddam and Van den Ende, 2012).

Regarding the “recovery” phenomenon, we analyzed the expression of the *SUC* genes together with four *SUS* genes and the cell wall invertase gene, *cwINV*, in leaves of plants recovered from the disease. When analyzed in R leaves, the expression of *SUC* transporters was higher than in leaves of D plants. Similarly, the gene encoding for the cell wall invertase was up-regulated both in D and R samples. On the other hand, the expression of all the SUS genes was found to be at the same level as leaves from H plants. This finding seems to suggest a direct relationship between phytoplasma and, in particular, SUSs that restore the level of expression of H plants when the pathogen disappears.

Recovered plants seem to fully establish the carbohydrate source function of leaves; moreover, recovered plants seem to acquire increased transport ability and defense signaling. This finding seems to be confirmed by the performance of field growing plants that become resistant to new attaches when recovered from phytoplasma diseases (Osler et al., 2000). Sugars such as glucose, fructose, and sucrose are recognized as signaling molecules in plants, in addition to their roles as carbon and energy sources (Koch, 2004; Rolland et al., 2006). Sucrose specifically stimulates the accumulation of anthocyanins (Solfanelli et al., 2006), which can act as antimicrobial agents in the plant defense system against pathogen invasion (Winkel-Shirley, 2001). Sugar signals, in particular a high sucrose:hexose ratio, may contribute to immune responses and probably function as priming molecules for more rapid and robust activation of defense to biotic or abiotic stress (Bolouri-Moghaddam and Van den Ende, 2012).

Stolbur is not detected in leaves of recovered grapevines, which is similar to observations in the crown of apple trees recovered from the phytoplasma-associated-Apple Proliferation disease (Osler et al., 2000; Carraro et al., 2004). For this reason, the signal triggering the up-regulation of sucrose transport and apoplastic cleavage must originate from organs distal from the leaves. Whereas the pathogen has been detected in roots of recovered apple trees (Carraro et al., 2004), to date no data are available for grapevine roots, probably because the stolbur titer is very low and diagnostic tools are still not sensitive enough. Concerning the features of the signal molecule, it is noteworthy that sugar production and use can be controlled in part at the transcriptional level by sugars themselves.

## Conclusion

Phytoplasmas interfere with plant processes, leading to severe changes in plant development and physiology, but to date the biochemical and molecular interactions between phytoplasmas and their hosts remain largely unexplored. “Recovery” from the disease is often observed, but the molecular basis is almost completely unknown as well.

The present work has unveiled structural and physiological modifications occurring in the grapevine leaf phloem, which is the site where phytoplasmas live, multiply and spread. Our findings have demonstrated that phloem is severely affected by phytoplasma infection. Callose accumulates anomalously in the infected sieve elements, but its deposition is only a part of the large number of modifications at the transcriptional level observed for sucrose transport and metabolism in the whole leaf. The decreased sucrose transport observed at the transcriptional level, as well as the increased sucrose cleavage mediated by cell wall invertase and SUS, confirm the establishment of a stolbur-induced carbohydrate sink in the leaves. Plants that have recovered from the disease seem to fully restore the carbohydrate source function of leaves and acquire increased transport ability and defense signaling.

## Conflict of Interest Statement

The authors declare that the research was conducted in the absence of any commercial or financial relationships that could be construed as a potential conflict of interest.

## Footnotes

1 http://frodo.wi.mit.edu/primer3/

2 http://www.genoscope.cns.fr/externe/GenomeBrowser/Vitis/

3 http://www.genoscope.cns.fr/externe/GenomeBrowser/Vitis/
